# Role of simultaneous fine needle aspiration cytology of palpable inguinal lymph node and primary tumor biopsy in carcinoma of the penis

**Published:** 2007

**Authors:** Nikhil Khattar, L. N. Dorairajan, Santosh Kumar

**Affiliations:** Department of Urology, Jawaharlal Institute of Postgraduate Medical Education and Research, Pondicherry - 605 006, India. E-mail: dorairajan_ln@hotmail.com

## COMMENTS

Inguinal lymphadenectomy is associated with significant morbidity. Hence, it cannot be advocated routinely in all patients with penile cancers as that will lead to unnecessary operations in 50% of patients with clinically palpable nodes and in 70-80% of patients with clinically negative inguinal nodes. At the same time, unlike other urological malignancies, lymphadenectomy alone can be curative in patients of carcinoma of the penis with positive nodes and should be performed. Thus, there has been ongoing effort to find ways to decrease the morbidity of lymphadenectomy and still provide treatment by making use of means for selecting patients at a higher risk for harboring a positive node. In a clinically negative groin, various experts have advocated the use of sentinel node biopsy, ultrasound-guided FNAC and medial inguinal lymph node biopsy. In the event of clinically palpable nodes, a course of antibiotics for 4-6 weeks after the treatment of the primary tumor, to separate significant lymphadenopathy from a reactive one, has been the standard practice. This leads to a considerable delay in providing definitive treatment to the patient for his groin nodes and the delay adversely affects the prognosis as compared to concomitant penile surgery and lymphadenectomy.[1]

The authors in this article have selected patients with clinically positive groin nodes before the treatment of the primary tumor and have subjected them to FNAC of the nodes, thus permitting a concomitant penectomy and groin dissection in those whose FNAC were positive. They noted that in 52% of patients, adopting this strategy avoided a six-week delay in providing definitive treatment to nodes. The authors, thus, endorse the recommendation of Horenblas[2] to treat groin lymph nodes early and abandon the waiting period of 4-6 weeks of antibiotic treatment as opposed to the recommendation of Senthil *et al* who advocate FNAC for palpable nodes after antibiotic therapy.[3] The authors have not mentioned the complications arising from the procedure. Although concomitant inguinal lymphadenectomy and primary tumor resection have been reported in earlier series to result in a mortality rate of 3-4%,[4] contemporary data do not show any mortality.[1] In view of this promising result, the authors' recommendation to perform early FNAC in palpable nodes and lymphadenectomy along with primary tumor surgery seems to be reasonable. The only matter of concern is the mention of a rare false-positive FNAC for tumor.

Although recommended in the algorithm that FNAC and excisional biopsy can be repetitively performed in FNAC-negative patients, the usefulness of this line of management has not been assessed in this study. As the false negative rate was only 7% in this study, it seems reasonable to manage the FNAC-negative cases along standard lines by reassessment after a six-week course of antibiotics instead of subjecting them to multiple procedures. However, there should be no doubt that if FNAC is negative, the patients have to be placed on a strict surveillance protocol.

## References

[1] Ornellas AA, Seixas AL, Marota A, Wisnescky A, Campos F, de Moraes JR (1994). Surgical treatment of invasive squamous cell carcinoma of the penis: Retrospective analysis of 350 cases. J Urol.

[2] Senthil Kumar MP, Ananthakrishnan N, Prema V (1998). Predicting regional lymph node metastasis in carcinoma of the penis: A comparison between fine-needle aspiration cytology, sentinel lymph node biopsy and medial inguinal lymph node biopsy. Br J Urol.

[3] Beggs JH, Spratt JS (1964). Epidermoid carcinoma of the penis. J Urol.

[4] Horenblas S (2001). Lymphadenectomy for squamous cell carcinoma of the penis. Part 1: Diagnosis of lymph node metastasis. BJU Int.

